# MiR224-3p inhibits hypoxia-induced autophagy by targeting autophagy-related genes in human glioblastoma cells

**DOI:** 10.18632/oncotarget.5871

**Published:** 2015-10-19

**Authors:** Xing Guo, Hao Xue, Xiaofan Guo, Xiao Gao, Shugang Xu, Shaofeng Yan, Xiao Han, Tong Li, Jie Shen, Gang Li

**Affiliations:** ^1^ Department of Neurosurgery, Qilu Hospital of Shandong University, Jinan, Shandong Province, P.R. China; ^2^ Brian Science Research Institute, Shandong University, Jinan, Shandong Province, P.R. China; ^3^ Department of Neurosurgery, Dezhou People's Hospital, Dezhou, Shandong Province, P.R. China

**Keywords:** hypoxia, autophagy, microRNAs, autophagy-related genes, glioblastoma

## Abstract

Human glioblastoma multiforme (GBM) is a malignant solid tumor characterized by severe hypoxia. Autophagy plays a protective role in cancer cells under hypoxia. However, the microRNA (miRNA)-related molecular mechanisms underlying hypoxia-reduced autophagy remain poorly understood in GBM. In this study, we performed a miRNA microarray analysis on GBM cells and found that numerous miRNAs were differentially expressed under hypoxic conditions. Further research showed that miR224-3p, one of the significantly down-regulated miRNAs, was involved in regulating hypoxia-induced autophagy in GBM cells. Overexpression of miR224-3p abolished hypoxia-induced autophagy, whereas knocking down endogenous miR224-3p increased autophagic activity under normoxia. In addition, we demonstrated that miR224-3p inhibited autophagy by directly suppressing the expression of two autophagy-related genes (ATGs), ATG5 and FAK family-interacting protein of 200 kDa (FIP200). Furthermore, *in vitro*, miR224-3p attenuated cell proliferation and promoted hypoxia-induced apoptosis, and *in vivo*, overexpression of miR224-3p inhibited tumorigenesis of GBM cells. Collectively, our study identified a novel hypoxia-down-regulated miRNA, miR224-3p, as a key modulator of autophagy by inhibiting ATGs in GBM cells.

## INTRODUCTION

Glioblastoma multiforme (GBM) is the most malignant and aggressive primary human brain tumor, with a uniformly poor prognosis [1]. Although therapeutic options have improved, GBM remains a difficult cancer to treat [2]. GBM contains a wide range of severely hypoxic regions. Hypoxia has emerged as a predominant feature of the solid tumor microenvironment, with a crucial role in tumor growth, progression and resistance to conventional cancer therapy [3]. Previous studies have illustrated that hypoxia-inducible factor (HIF) regulates the responses of tumor cells to hypoxia by controlling the transcription of hundreds of target genes [4]. However, our understanding of hypoxia-regulated molecular mechanisms in GBM remains limited.

Hypoxia can affect mRNA transcription, mRNA stability, and protein stability. Additionally, it induces a distinct shift in a specific group of miRNAs [5]. MiRNAs are 17–22-nucleotide, noncoding and single-stranded RNA molecules that regulate gene expression by blocking mRNA translation and/or mediating mRNA degradation [6, 7]. Notably, miRNAs have also been implicated in the modulation of autophagic activity. Previous studies have demonstrated that, under hypoxic conditions, miRNAs [8–11] can modulate a series of autophagy-promoting genes at different stages of autophagy to mediate autophagosome formation. Hypoxia-induced autophagy in tumor cells can lead to treatment resistance and malignant progression [12]. Those facts led us to hypothesize that hypoxia enhances autophagy by deregulating miRNAs. However, it has not been clearly established whether hypoxia-regulated miRNAs modulate autophagy in GBM cells. A better understanding of miRNA-related mechanisms may be critical for the development of new GBM therapies and the supplementation of current GBM therapies.

Autophagy is an evolutionarily conserved cellular catabolic process involving self-digestion, elimination and turnover of intracellular proteins and organelles via delivery to lysosomes [13]. Autophagy-related genes (ATGs) are essential to drive this cellular process and are directly regulated by multiple miRNAs [14]. To date, 31 ATGs have been described [15]. Among these, one subset is essential for autophagosome formation and is referred to as the “core” molecular machinery. Several important ATG complexes act in concert during autophagosome formation, including the unc-51-like autophagy-activating kinase 1 (ULK1) complex and the ATG12-ATG5-ATG16L1 conjugation system [16]. Autophagy induction is initiated by the ULK1 complex, which contains ULK1 (ATG1), ATG13 and FAK family-interacting protein of 200 kDa (FIP200). Microtubule-associated protein 1 light chain 3 beta (LC3B) conjugation to the lipid phosphatidylethanolamine (PE) necessary for autophagic membrane elongation and vesicle completion requires the activity of the ATG12-ATG5-ATG16L1 complex [17].

In this study, we performed a hypoxic miRNA microarray and focused on the signature of significantly hypoxia-dysregulated miRNAs in human GBM cell lines. Through screening, we found that miR224-3p, the most significantly down-regulated miRNA, played an inhibitory role in the regulation of autophagy in GBM cells. MiR224-3p down-regulation enhances hypoxia-induced autophagy by releasing the expression of target genes, including ATG5 and FIP200. Our study highlights the relationship between hypoxia, dysregulated miRNAs and autophagy in GBM and identifies a novel miRNA that modulates autophagy.

## RESULTS

### Autophagic activity is activated under hypoxia in human glioblastoma cell lines

**Figure 1:**
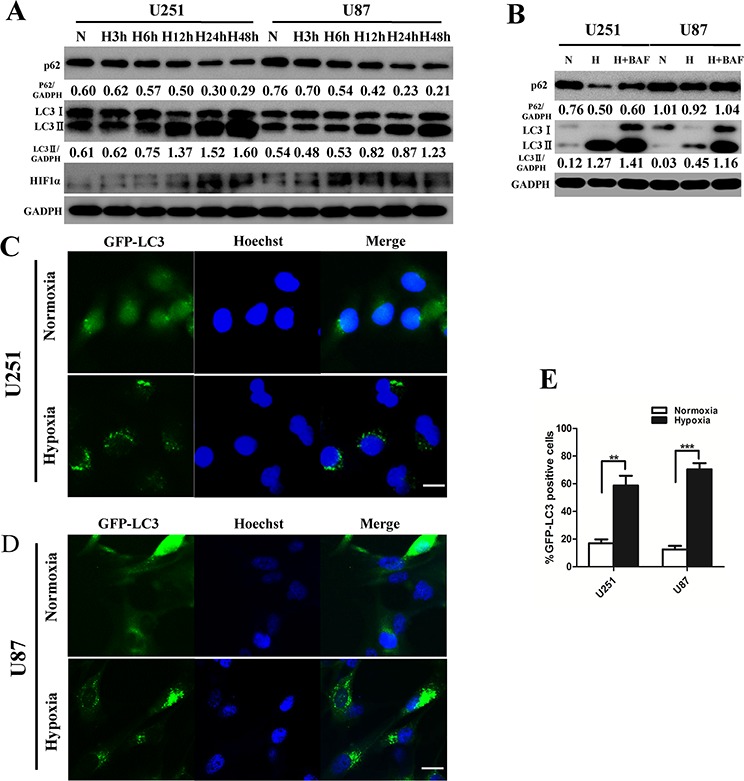
Hypoxia induces glioblastoma cell autophagic activity **A.** U251 and U87 cells were exposed to hypoxia (1% O_2_) for 3 h, 6 h, 12 h, 24 h, or 48 h. LC3B, p62 (SQSTM1) and GAPDH levels were determined by Western blot. **B.** U251 and U87 cells were cultured under hypoxia for 24 h with or without BAF (20 nM). Protein expression levels were analyzed by Western blot. **C.** and **D.** U251 and U87 cells stably expressing GFP-LC3 were subjected to hypoxia for 24 h and then fixed. The nuclei were stained by Hoechst. Representative images are shown. Scale bar, 20 μm. **E.** Quantitative analysis of the rates of GFP-LC3 puncta-positive cells in (C) and (D) are shown. At least 200 cells in (C) and (D) were examined in each experimental group. The data shown are the mean ± SD of independent experiments, *n* = 3. **P < 0.01, ***P < 0.001, Student's 2-tailed *t* test.

Autophagy is induced in tumor cells within hypoxic tumor regions [18]. To investigate the autophagic activity under physiological hypoxic conditions (1% oxygen) in GBM cells, we performed LC3B conversion and GFP-LC3 puncta-formation assays. First, we examined the expression of LC3B-II by Western blot in U251 and U87 cells. Culturing U251 and U87 glioma cell lines in hypoxia for 3, 6, 12, 24 or 48 h increased LC3B-II accumulation in a time-dependent manner (Figure 1A). The conversion of LC3B-I to LC3B-II indicates the formation of autophagosomes [19]. Quantitative assessment of the ratio of LC3B-II to GADPH serves as the primary indicator of autophagy induction [20, 21]. Next, U251 and U87 cells that stably expressed GFP-LC3 proteins were exposed to hypoxia for 24 h, and GFP-LC3 localization was examined by fluorescence microscopy. GFP-LC3 puncta appeared in the cytoplasm, reflecting the recruitment of LC3B proteins to autophagosomes [22]. There was a significant increase in GFP-LC3 puncta-positive cells under hypoxia (Figure 1C, 1D, 1E). Consistent with the LC3B conversion assay, the quantification of the percentage of GFP-LC3 puncta-positive cells confirmed that hypoxia induced autophagosome accumulation in both U251 and U87 cells. Thus, both assays suggested that hypoxia induces autophagosome accumulation in human GBM cells.

To distinguish whether LC3B-II accumulation is due to autophagy induction or to a block in downstream steps, we performed autophagic flux assays. Sequestosome 1 (SQSTM1/p62), a polyubiquitin-binding protein, is selectively incorporated into autophagosomes through direct binding to LC3B and efficiently degraded during autophagy. Thus, the total cellular levels of SQSTM1 reflect autophagic activity [23]. The late autophagy inhibitor bafilomycinA1 (BAF) blocked hypoxia-induced p62 degradation in U251 and U87 cells. BAF treatment significantly increased LC3B-II levels under hypoxia (Figure 1B). These data demonstrate that hypoxia induces the autophagic activity of human GBM cells.

### Hypoxia induces miR224-3p down-regulation in glioblastoma cell lines, and miR224-3p expression is low in human glioma

**Figure 2:**
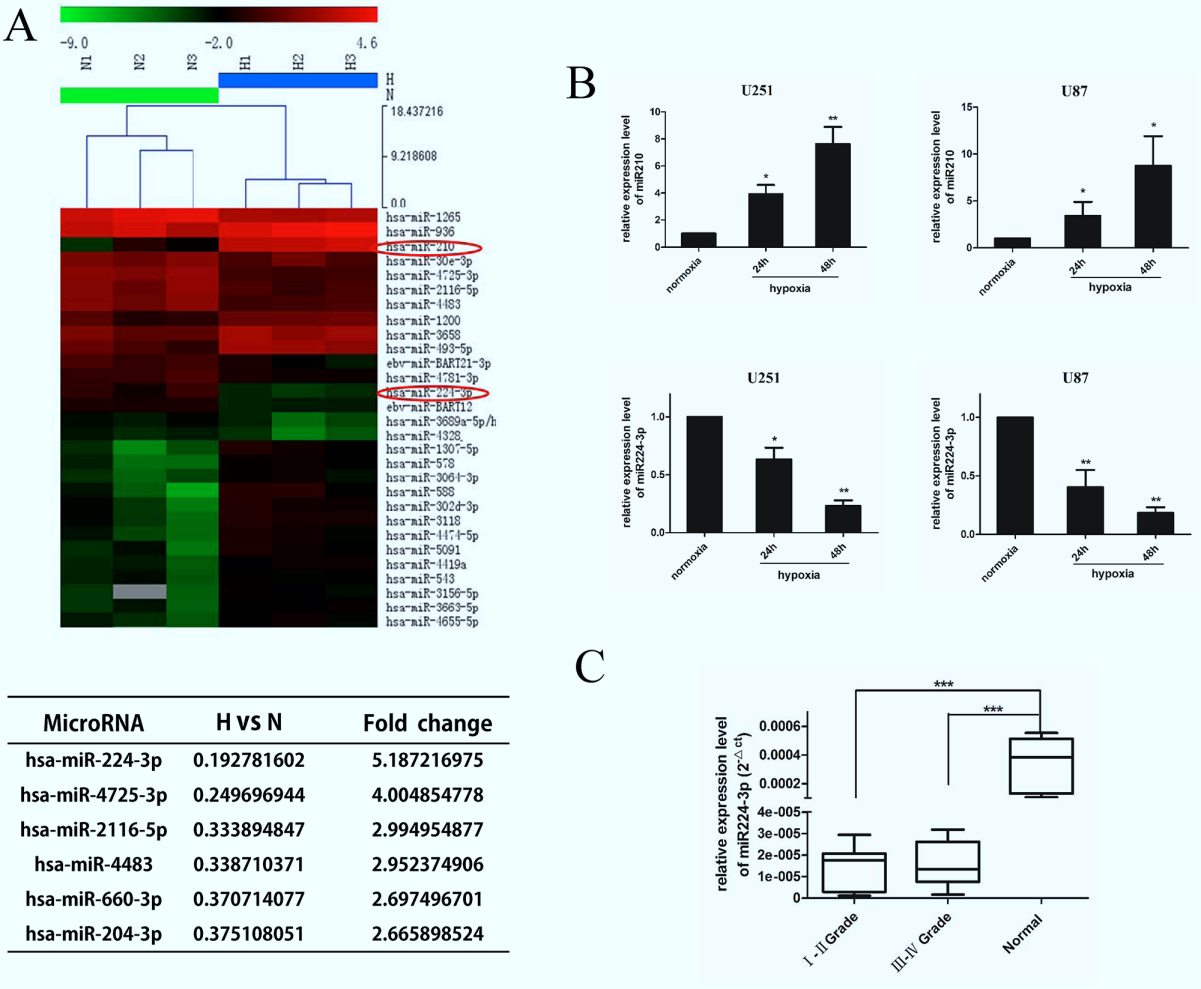
miR224-3p is down-regulated under hypoxia in glioblastoma cell and expressed at low levels in human glioma **A.** U251 cells were exposed to hypoxia for 24 h and collected for miRNA microarray. The hypoxic glioblastoma miRNA assay is shown as a heat map (in part). Up-regulated miRNAs are shown in red, while down-regulated microRNAs are shown in green. The hypoxia markers miR210 and miR224-3p are tagged by a red circle. The six down-regulated miRNAs with at least a 2.65-fold expression change are listed. **B.** U251 and U87 cells were exposed to hypoxia (1% O_2_) for 24 h and 48 h. Cells were collected for q-PCR to quantify miR224-3p expression. The data shown are the mean ± SD of independent experiments, *n* = 3. **C.** miR224-3p expression in glioma and normal brain tissues was determined by q-PCR analysis and grouped according to WHO I, II grade (*n* = 14), III, IV grade (*n* = 16) and normal brain tissue (*n* = 6). The boxes represent the lower and the upper quartiles with medians; the whiskers illustrate the 10 to 90 percentiles of the samples. *P < 0.05, **P < 0.01, ***P < 0.001, Student's 2-tailed *t* test or one-way ANOVA.

Recently, several lines of evidence have directly established miRNAs as key elements in the molecular response of tumor cells to hypoxia. To further understand the miRNA signature of GBM cells under hypoxia, we identified differentially expressed miRNAs using a miRNA microarray (ArrayExpress accession number: E-MTAB-3886). In total, 84 miRNAs were differentially expressed (Supplementary Figure S2A, shown as a Volcano plot), including eight up-regulated *homo sapiens* (*hsa*)-miRNAs with at least a 5-fold expression change and six down-regulated *hsa*-miRNAs with at least a 2.65-fold expression change (Figure 2A). The hypoxic marker miR210 [24] was up-regulated more than 30-fold, confirming the effective hypoxic levels of our GMB cells culture conditions. We hypothesized that the significantly dysregulated miRNAs contributed to the hypoxia-induced autophagy modulation of GBM cell lines. To confirm our hypothesis, we overexpressed the six significantly down-regulated miRNAs by transfecting U251 cells with miRNA mimic under hypoxia. We then performed an LC3 conversion assay by Western blot. The transfection efficiency was evaluated by FAM transfection and quantitative real-time PCR (q-PCR) (Supplementary Figure S1A, S1B). Interestingly, there was a significant decrease in LC3B-II in miR224-3p-transfected cells (Supplementary Figure S2B). Accordingly, we focused on the inhibitory effects of hypoxia-down-regulated miR224-3p on the autophagic activity of GBM cells.

To further validate the expression of miR224-3p, we measured miR224-3p expression in U251 and U87 cells under hypoxic conditions at 24 h and 48 h by q-PCR. The expression levels of miR210 increased under hypoxic culture conditions (Figure 2B, upper panel), indicating effective hypoxia. In contrast, the expression levels of miR224-3p were low under normoxic culture conditions. When exposed to hypoxia, miR224-3p was significantly down-regulated in a time-dependent manner in both GBM cell lines (Figure 2B, lower panel). At 48 h after hypoxia treatment, miR224-3p expression decreased more than 5-fold. The consistency between the miRNA microarray data and the results of the q-PCR assay demonstrate the validity of the microarray.

To evaluate the clinical significance of miR224-3p, thirty glioma specimens [sixteen high-grade tissues (World Health Organization (WHO); WHO III-IV) and fourteen low-grade tissues (WHO I-II)] and six normal brain specimens were collected to detect miR224-3p expression by q-PCR. MiR224-3p was down-regulated in human glioma tissues compared with normal brain tissues (*P* < 0.001). There was no significant difference between expression in high-grade glioma and low-grade glioma (Figure 2C). Therefore, we propose that miR224-3p potentially inhibits hypoxia-induced autophagy and is expressed at low levels in human glioma.

### MiR224-3p influences glioblastoma cell autophagic activity

**Figure 3:**
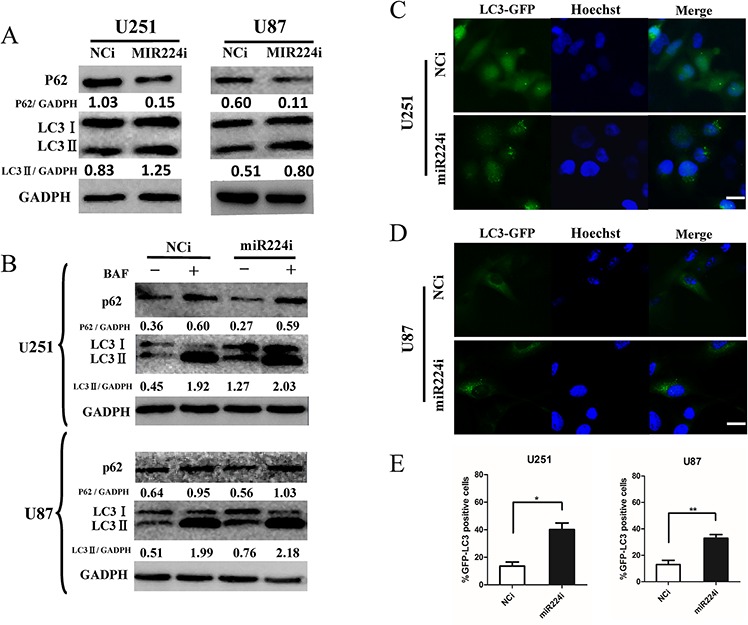
miR224-3p influences glioblastoma cell autophagic activity **A.** U251 and U87 cells were transfected with miR224i (100 nM) for 24 h. LC3B, p62 and GAPDH levels were determined by Western blot. **B.** U251 and U87 cells were transfected with miR224i (100 nM) or NCi and BAF (20 nM) were applied to the medium for 24 h at 24 h post transfection. LC3B, p62 and GAPDH levels were determined by Western blot. **C.** and **D.** U251 and U87 cells stably expressing GFP-LC3 were transfected with miR224i or NCi for 24 h and then fixed. Representative images are shown. Scale bar, 20 μm. **E.** Percentages of GFP-LC3 puncta-positive cells in (C) and (D) are shown. The data shown are the mean ± SD of independent experiments, *n* = 3. MiR224i, miR224-3p inhibitor; NCi, miRNA inhibitor negative control. *P < 0.1, **P < 0.01, Student's 2-tailed *t* test.

After screening the hypoxia GBM cell miRNA microarray, we detected miR224-3p as a novel autophagy-related miRNA. To precisely explore the role of miR224-3p in autophagic activity, we repeated LC3 conversion and GFP-LC3 puncta-formation assays in both U251 and U87 cell lines. MiR224-3p inhibitors used to inhibit the level of endogenous miR224-3p were transfected into U251 and U87 cells. The expression of LC3B-II increased and that of p62 decreased (Figure 3A), suggesting that the miR224-3p inhibitor enhanced autophagy in the transfected cells. At the same time, we also examined the location of GFP-LC3 by fluorescence microscopy in miR224-3p inhibitor-transfected U251 and U87 cells stably expressing the GFP-LC3 fusion protein. There was a significant increase in GFP-LC3 puncta in miR224-3p inhibitor-transfected cells compared with the negative control cells (Figure 3C, 3D, 3E). In the same way, miRNA224-3p mimic was transfected into both cell lines, and autophagy was slightly inhibited, as indicated by the decreased LC3B-II expression and increased accumulation of p62 (Supplementary Figure S3B).

Finally, we performed an LC3 turnover assay in both miR224-3p mimic-transfected and inhibitor-transfected cells to exclude the possibility of a block in the downstream steps during autophagic flux. GBM cells were treated with the lysosomotropic reagent BAF to block autophagic degradation. BAF treatment significantly increased LC3B-II in both miR224-3p mimic- and inhibitor-transfected cells. In addition, the miR224-3p inhibitor group exhibited higher levels of LC3BII compared with the NCi group (Figure 3B). In contrast, the accumulation of LC3B-II in the miR224-3p mimic group was lower than in the control group after BAF treatment (Supplementary Figure S3B). These data suggest that miR224-3p indeed affected autophagic activity in the GBM cell lines. Furthermore, knockdown of endogenous miR224-3p highly induced GBM cell autophagic activity.

### Overexpression of miR224-3p inhibits hypoxia-induced autophagy in glioblastoma cells

**Figure 4:**
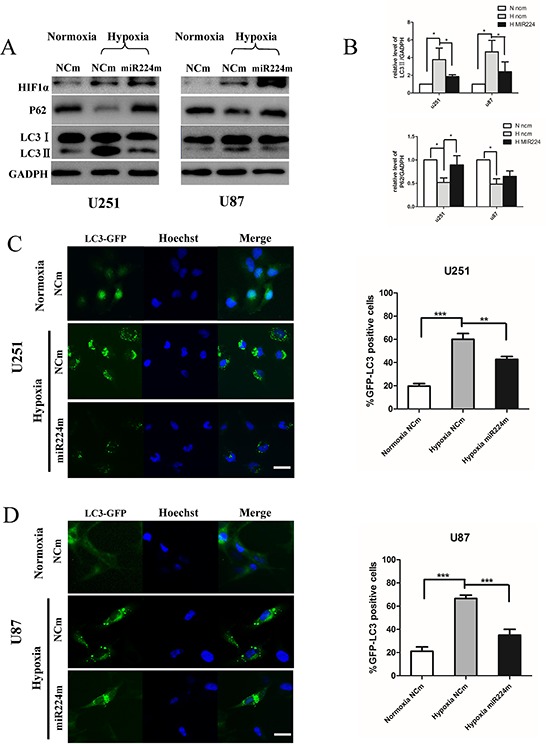
Overexpression of miR224-3p inhibits hypoxia-induced autophagy **A.** U251 or U87 cells transfected with miR224m or NCm for 48 h were cultured in 1% O_2_ for another 24 h. Cells were then harvested for Western blot. **B.** The relative ratios of p62 (SQSTM1)/GAPDH and LC3B-II/GAPDH were calculated following Quantity One analysis. The data shown are the mean ± SD of independent experiments, *n* = 3. **C, D.** U251 and U87 cells stably expressing GFP-LC3 were transfected with miR224m or NCm for 48 h, exposed to 1% O_2_ for another 24 h and then fixed. Representative images from the quantification are shown. Scale bar, 20 μm. Quantitative analyses of rate of GFP-LC3 puncta-positive cells in (C) and (D) are shown. The data shown are the mean ± SD of independent experiments, *n* = 3. MiR224m, miR224-3p mimic; NCm, miRNA mimic negative control. *P < 0.05, **P < 0.01, ***P < 0.001 Student's 2-tailed *t* test or one-way ANOVA.

**Figure 5:**
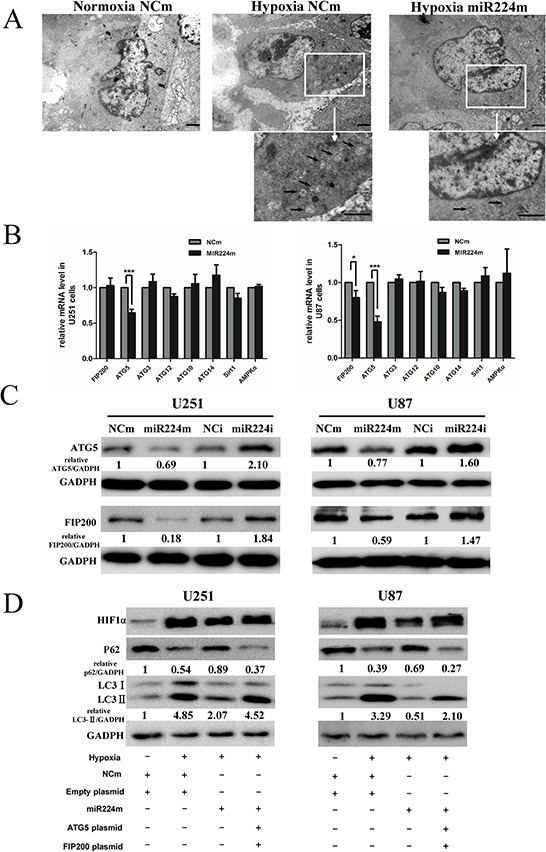
miR224-3p regulates autophagy by targeting multiple ATGs **A.** U251 cells transfected with miR224m or NCm were exposed to hypoxia for 24 h. Characteristic autophagosomes (arrows) were shown in representative images from transmission electron microscopy. Scale bar, 1.2 μm. **B.** U251 and U87 cells were transfected with miR224m or NCm and incubated for 48 h. mRNA expression levels of predicted targets of miR224-3p were determined by q-PCR. The data shown are the mean ± SD of independent experiments, *n* = 3. **C.** U251 and U87 cells were transfected with miR224m, NCm or miR224i, NCi and incubated for 48 h. The cells were then harvested for Western blot. **D.** U251 and U87 cells were transfected with miR224m and co-transfected with both ATG5 and FIP200 plasmids at the same time for 24 h and then exposed to the hypoxia. After 24 h, cells were harvested for Western blot. Quantitative relative analyses of the western blot were calculated following Quantity One analysis. MiR224m, miR224-3p mimic; miR224i, miR224-3p inhibitor; NCm, miRNA mimic negative control; NCi, miRNA inhibitor negative control. *P < 0.05, ***P < 0.001, Student's 2-tailed *t* test.

MiR224-3p is down-regulated under hypoxia, indicating that miR224-3p plays an important role in inhibiting the hypoxia-induced autophagy of GBM cells. To document the effects of miR224-3p on hypoxia-induced autophagy, we overexpressed miR224-3p in both U251 and U87 cells to reverse its low levels during hypoxia treatment and repeated the above validation assays. Both LC3B-II and p62 expression were measured by Western blot. Compared with the negative control, LC3B-II expression and p62 degradation during hypoxia treatment were markedly reduced upon miR224-3p mimic transfection (Figure 4A, 4B). At the same time, hypoxia-induced GFP-LC3 puncta accumulation was dramatically suppressed by transfecting miR224-3p mimic into U251 and U87 cells that stably expressed GFP-LC3, reflecting a decrease in autophagic activity (Figure 4C, 4D). To further evaluate the repressive effects of the miR224-3p on hypoxia-induced autophagy, transmission electron microscopy was used to visualize autophagosomes in U251 cells. Transmission electron microscopy revealed more characteristic autophagosomes in U251 cells exposed to hypoxia than that in cells under normoxia and transfected miR224-3p mimics under hypoxia (Figure 5A). These results demonstrate the crucial importance of decreasing the expression of endogenous miR224-3p in regulating autophagy under hypoxia.

### MiR224-3p regulates autophagy by targeting ATGs in glioblastoma cells

As we had established the vital role of miR224-3p in the hypoxia-induced autophagy of GBM cells, we next evaluated the effects of miR224-3p on potential targets related to autophagy. We used predicted miRNA target databases including “miRanda”, “DIANA-mT”, “miRDB” and “TargetMiner” to search for potential targets of miR224-3p, and multiple target genes were found. To reduce the number of target genes, we performed a Western blot assay to distinguish whether miR224-3p inhibited hypoxia-induced autophagy by targeting the ERK/AKT/mTOR pathway, which is the main regulator of autophagy. MiR224-3p mimic and inhibitor were transfected into both GBM cell lines, which were then harvested for the assay. We also used phosphorylation site-specific antibodies to directly measure the activation of mTOR, AKT and ERK1/2. There were no pronounced differences in the expression of ERK1/2, p-ERK1/2, AKT, p-AKT and p-mTOR between the miR224-3p mimic and negative control mimic groups. Similarly, there were no changes in the ERK/AKT/mTOR pathway between the miR224-3p inhibitor and negative control inhibitor groups (Supplementary Figure S2C).

After excluding potential targets in the ERK/AKT/mTOR pathway, we focused on targets involved in the molecular mechanisms of autophagy. We chose target genes predicted in at least three of the above-mentioned databases, including ATG5, ATG3, ATG10, ATG12, ATG14 and FIP200. Moreover, SIRT1 and AMPK1α were also putative direct targets of miR224-3p. We then examined the mRNA levels of the putative miR224-3p targets by q-PCR in GBM cells transfected with miR224-3p mimic. In both U251 and U87 cells, the transfection of miR224-3p mimic, but not the negative control mimic, significantly reduced ATG5 mRNA levels. FIP200 mRNA levels decreased only in U87 cells. However, other predicted miR224-3p target genes did not show significant changes in either U251 or U87 cells (Figure 5B).

miRNAs regulate gene expression either by blocking mRNA translation or by mediating mRNA degradation. Although we evaluated the mRNA levels of putative target genes, the target protein levels needed to be confirmed. Consistent with the alteration of ATG5 mRNA levels, transfection of the miR224-3p mimic and inhibitor resulted in the reduction and increase, respectively, of ATG5 protein levels. In addition, the changes in FIP200 protein levels suggested that FIP200 is also a miR224-3p target (Figure 5C). Western blotting with specific antibodies showed that the SIRT1 and ATG3 protein levels had not decreased in miR224-3p-overexpressing cells (Supplementary Figure S2D). Therefore, at both the mRNA and protein levels, we demonstrated that miR224-3p regulates autophagy independent of the ERK/AKT/mTOR pathway, SIRT1 and ATG3.

To further define the involvement of ATG5 and FIP200 in the suppression of autophagy by miR224-3p, both ATG5 and FIP200 overexpressing-plasmids were co-transfected into miR224-3p-overexpressing U251 and U87 cells. The transfection efficiency was confirmed by Western blot (Supplementary Figure S1C). Next, we performed an LC3B conversion assay to evaluate the autophagic activity changes in these cells. Both ectopic expression of ATG5 and FIP200 significantly rescued miR224-3p-induced inhibition of autophagy (Figure 5D). Our data demonstrated that miR224-3p suppresses the expression of two ATGs, including ATG5 and FIP200. Thus, subsequent studies will concentrate on ATG5 and FIP200.

### MiR224-3p directly targets ATG5 and FIP200 by interacting with 3′ UTRs

**Figure 6:**
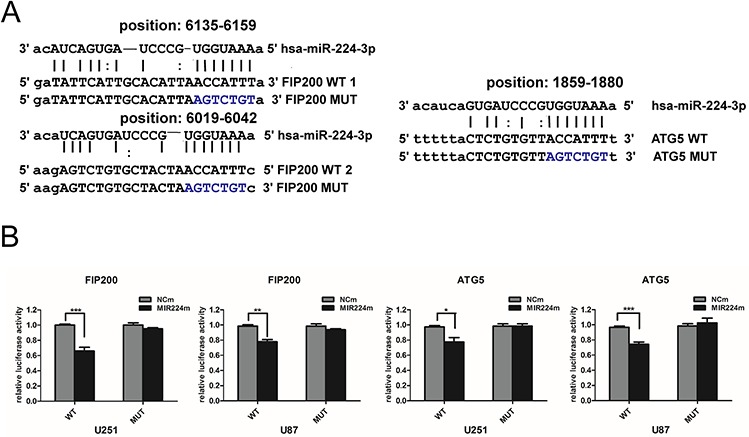
miR224-3p directly targets both ATG5 and FIP200 **A.** Predicted binding sequences for miR224-3p and matches in the ATG5 and FIP200 3′ UTRs. **B.** Dual-luciferase reporter vectors containing the wild-type or mutated 3′ UTR fragments of ATG5 and FIP200 cloned into the pmirGLO-luciferase plasmids. Luciferase reporter assayed at 48 h after U251 cell transfection with wild-type (WT) or mutated (MUT) plasmids, co-transfected with miR224m, NCm. The data shown are the mean ± SD of independent experiments, *n* = 3. MiR224m, miR224-3p mimic; NCm, miRNA mimic negative control. *P < 0.05, **P < 0.01, ***P < 0.001, Student's 2-tailed *t* test.

To further determine whether miR224-3p regulates target genes via direct interactions with binding sites in their 3′ untranslated regions (UTRs), we performed a dual-luciferase reporter assay. We used the miRanda, DIANA-mT and TargetMiner databases to predict binding sequences with a high possibility of ranking in the 3′ UTRs of target genes (Figure 6A). Then, we designed pmirGLO-luciferase plasmids containing either the wild-type (WT) or mutated (MUT) miR224-3p binding sequences in the 3′ UTRs of ATG5 and FIP200. The pmirGLO-luciferase plasmids were co-transfected with either miR224-3p mimic or negative control mimic. Luciferase activity was determined 48 h after transfection. MiR224-3p overexpression significantly inhibited luciferase activity in the wild-type ATG5 and FIP200 3′ UTRs but not in the mutated 3′ UTR plasmids, demonstrating the specificity of the miR224-3p binding sites in their 3′ UTRs (Figure 6B). Thus, it is not unexpected that miR224-3p regulates the hypoxia-induced autophagy of GBM cells by directly targeting pleiotropic ATGs.

### MiR224-3p expression levels correlate with ATG5 and FIP200 expression in glioma tissues

**Figure 7:**
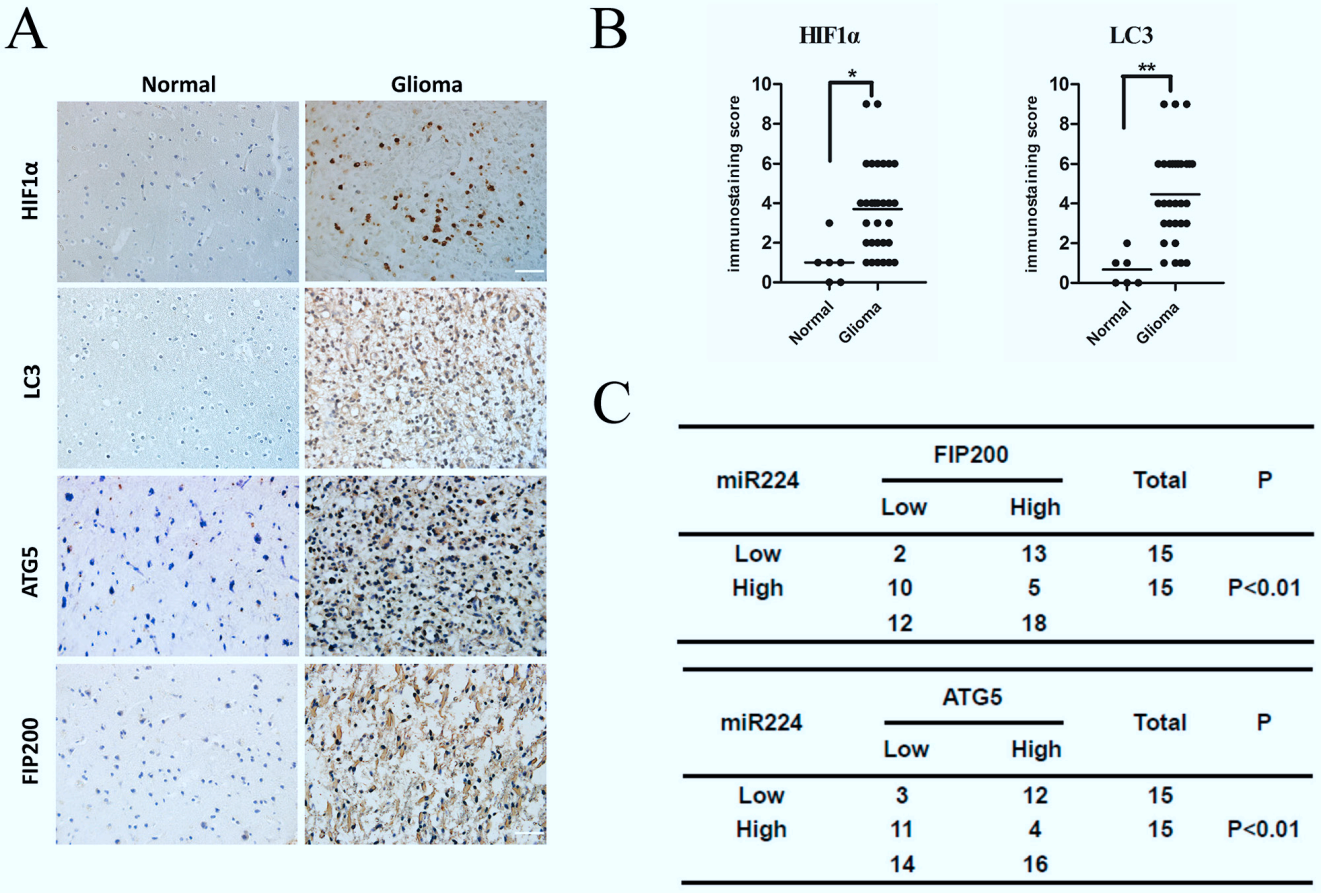
miR224-3p inversely correlates with ATG5 and FIP200 expression in human glioma tissues **A.** Representative images of glioma and normal brain tissue showed the immunostaining of HIF1α, LC3B, ATG5 and FIP200 (magnification, 400×). **B.** The total immunostaining scores of HIF1α and LC3B were estimated. The significant differences in expression between glioma and normal brain were analyzed by Student's 2-tailed *t* test. **C.** Associations between ATG5 and miR224-3p, FIP200 and miR224-3p expression in 30 glioma tissue specimens were analyzed by Fisher's exact test. Patients were classified as high or low miR224-3p expression groups based on the miR224-3p-expression in tumors above or below the median values respectively. *P < 0.05, **P < 0.01.

To test the clinical relevance of the above observations, we investigated the expression of HIF1a, LC3B, ATG5 and FIP200 in clinical specimens. We found that glioma tissues exhibited stronger immunohistochemical signals for both HIF1a and LC3B compared with normal tissues (Figure 7A, 7B). This result demonstrated that glioma tissues exhibit high autophagy activity when they remain under hypoxic conditions. In addition, we also showed that glioma tissues expressed higher levels of ATG5 and FIP200 (Figure 7A). Furthermore, we observed that miR224-3p levels inversely correlated with the protein levels of ATG5 and FIP200 (*P* < 0.01) (Figure 7C), which definitely indicated that miR224-3p regulates ATG5 and FIP200 in glioma tissues. Taken together, our results suggest that miR224-3p plays an important role in the regulation of hypoxia-induced autophagy in glioma tissues.

### MiR224-3p attenuates glioblastoma cell proliferation and induces hypoxia-induced apoptosis

**Figure 8:**
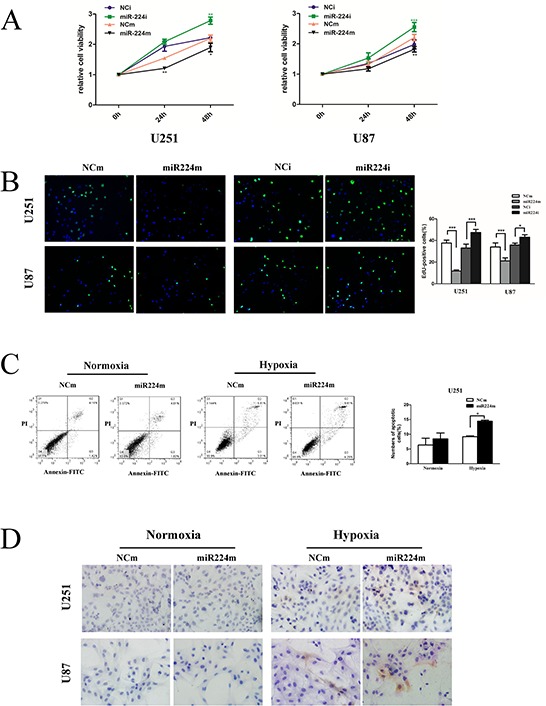
miR224-3p suppresses glioblastoma cell proliferation and promotes hypoxia-induced apoptosis **A.** U251 and U87 cells were transfected with 100 nM miR224m, NCm or miR224i, NCi for 24 h, and CCK8 assays were then performed at each time point (0 h, 24 h, 48 h). **B.** Proliferation of transfected U251 and U87 cells as in (A) was examined by EdU assay. **C.** miR224-3p overexpression was performed for 24 h, and then U251 cells were incubated under hypoxia and normoxia for 48 h. Transfected cells were fixed and stained with propidium iodide (PI) and FITC for flow cytometry to measure the apoptosis rate. The data shown are the mean ± SD of independent experiments, *n* = 3. **D.** U251 and U87 cells treated as (C) were seeded on the glass slide and fixed. Cell immunohistochemistry was performed to detect apoptosis using an anti-cleaved caspase-3 antibody. MiR224m, miR224-3p mimic; miR224i, miR224-3p inhibitor; NCm, miRNA mimic negative control; NCi, miRNA inhibitor negative control. *P < 0.05, **P < 0.01, ***P < 0.001, Student's 2-tailed *t* test.

Recent studies have strongly implicated that autophagy correlates with proliferation and favors cell survival under hypoxia by reducing apoptosis [25–27]. To further understand the biology of miR224-3p in GBM, we investigated the effects of miR224-3p on GBM cell proliferation and hypoxia-induced apoptosis. We observed that miR224-3p overexpression attenuated cell proliferation in both U251 and U87 cells, and neutralizing the endogenous miR224-3p improved proliferation (Figure 8A, 8B). We also examined the effects of miR224-3p on cell apoptosis both under hypoxia and normoxia. Transfection of miR224-3p mimic did not change the rate of apoptotic cells (Figure 8C) or the activity of caspase3 (Figure 8D) under normoxia. In contrast, under hypoxia, miR224-3p significantly promoted the apoptosis of GBM cells compared with the NCm group (Figure 8C, 8D).

**Figure 9:**
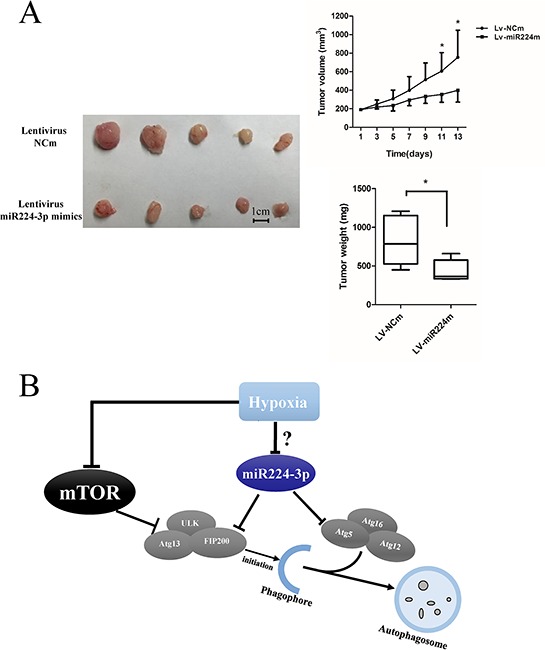
miR224-3p inhibits growth of GBM *in vivo* **A.** U87 cells were injected into mice subcutaneously. After the tumor was established, 20 μl of lentivirus-miR224m or lentivirus-NCm was injected into tumors twice a week followed by monitoring of tumor size for 2 weeks. U87 cell xenografts were measured by tumor volume, tumor weight. The data shown are the mean ± SD, *n* = 5. **B.** Schematic diagram of miR224-3p regulation of hypoxia-induced autophagy. MiR224-3p targets the expression of ATG5 and FIP200, which in turns suppresses the formation of autophagosomes. MiR224m, miR224-3p mimic; NCm, miRNA mimic negative control. *P < 0.05, Student's 2-tailed *t* test.

To extend our findings, we established a glioma mouse xenograft model using U87 cells. After tumor formation, we carried out intratumoral injections of miR224-3p mimic-expressing lentivirus. The transfection efficiency of the lentivirus was validated at the cell level (Supplementary Figure S1D). There were significantly smaller volumes of subcutaneous tumors in the miR224-3p mimic group compared with the control group (Figure 9A). Taken together, our data show that miR224-3p demonstrated antioncogenic activities in glioma.

## DISCUSSION

Hypoxia is a major feature of solid tumors due to inadequate blood supply, especially in rapidly growing tumors such as GBM [28]. The molecular mechanisms responsible for the hypoxia-induced tumor malignancy progression have not been fully revealed, but increasing evidence has implicated miRNAs in the regulation of hypoxia [29–31]. More importantly, enhanced autophagy enables cell survival by maintaining energy production that leads to tumor growth and therapeutic resistance under hypoxia. The increasing appreciation for miRNAs in hypoxia-induced autophagy has focused attention on significantly dysregulated miRNAs. In this study, we screened a miRNA microarray of GBM cells cultured under hypoxic conditions and identified hypoxia-down-regulated miR224-3p as a novel inhibitor of autophagy in GBM. By directly suppressing the expression of the critical ATGs, ATG5 and FIP200, miR224-3p inhibits the autophagic activity of GBM. In addition, we discovered that overexpression of miR224-3p attenuates GBM cell proliferation and promotes hypoxia-induced apoptosis. Moreover, miR224-3p confers anti-tumorigenicity abilities on GBM cells *in vivo*.

The predominant role of HIF in the response to hypoxia by controlling the coordination of transcriptional changes [32] has now gained wide acceptance. Extensive studies have shown that HIF augments the transcription of multiple miRNAs through direct binding to hypoxia response elements (HREs) [33, 34]. Some of the up-regulated miRNAs have previously been shown to be involved in autophagy activity [35]. In contrast, hypoxia also selectively represses certain miRNAs that respond to hypoxia through less well-characterized mechanisms, including EGFR [36] and transcription factors (TFs), such as NF-κB, SPI1 and p53 [37]. Bandara found that hypoxia represses miRNA biogenesis proteins in breast cancer cells [38]. A recent study by Sun *et al.* demonstrated that miR20a is a hypoxia-down-regulated miRNA that regulates autophagy by targeting ATG16L [39]. In our previous study, we hypothesized that hypoxia significantly down-regulates miR224-3p in response to increase autophagic activity. We examined the expression of miR224-3p, HIF1α and LC3B in clinical glioma specimens and normal brain tissues. The consistency between the lower expression of miR224-3p and the higher expression of HIF1α and LC3B in glioma tissue in part validates the hypothesis. Here, we used DMOG, a HIF1α stabilizer, in normoxia to examine the effects of HIF1α on the expression of miR224-3p. The results demonstrated that miR224-3p was down-regulated independent of HIF (Supplementary Figure S3A). However, the specific mechanism by which hypoxia down-regulates miR224-3p requires further investigation.

MiRNA could target multiple genes and has functions in a variety of pathophysiologic processes [40]. We confirmed that miR224-3p targeted both of ATG5 and FIP200. ATG5 and FIP200 are core molecular machinery components involved in autophagosome formation [41]. In our study, miR224-3p attenuated ATG5 mRNA and protein levels in both U251 and U87 transfected cells. However, the mRNA levels of FIP200 were only down-regulated in U87 transfected cells. The lower expression of FIP200 in U251 cells may explain this phenomenon, which was verified by protein expression through Western blotting. More importantly, the effects of miR224-3p on ATG5 and FIP200 were direct, as miR224-3p-response elements (MREs) were found in the 3′ UTRs of the ATG5 and FIP200 genes. In this paper, we also found that ATG5 and FIP200 were more highly expressed in clinical glioma tissues compared with normal brain tissue. Furthermore, both targets inversely correlated with the expression of miR224-3p in glioma specimens. These findings supported our own observations from a clinical perspective.

ATG5 has previously been reported to be the mutual target of different miRNAs to adjust autophagy. For instance, miR-181a and miR30a have been reported to inhibit autophagy in cancer cells by down-regulating ATG5 [42, 43]. A recent study of prostate cancer demonstrated that the up-regulated expression of ATG5 might play a role in tumorigenesis [44]. Moreover, the up-regulated expression of ATG5 in cancers provides further evidence to indicate the potential role of down-regulated miR224-3p in tumorigenesis.

Prior to this, there have been no studies of miRNAs targeting FIP200. Because FIP200 was identified as a mammalian homologue of ATG17 [45] and was essential for autophagosome formation, the physiological function of FIP200 *in vitro*, *in vivo* and in clinical specimens had been reported in glioma and other tumors [26, 46]. Those studies indicated that FIP200 expression is limited in normal brain tissues. In contrast, FIP200 was detected in tumor cells. Consistent with our findings, FIP200 expression in glioma and brain tissues is inversely associated with miR224-3p levels. Recently, in a Drosophila study, overexpression of FIP200 enhanced autophagy in an Atg1-dependent manner [34], which may explain why knocking down endogenous miR224-3p increased autophagic activity in normoxia. Autophagy has dual roles in cancer [47], and whether autophagy protects against or causes cancer remains unclear. Recent studies have revealed that the inhibition of autophagy by FIP200 ablation suppresses mammary tumor initiation and progression, and Wang *et al*. established the prognostic significance of ATG5 and FIP200 in patients with breast cancer [48]. Furthermore, miR210, one of the most significantly up-regulated miRNAs in hypoxia, can identify early systemic metastasis recurrence in melanoma patients [49]. These data allowed us to hypothesize that, in glioma, hypoxia-regulated miR224-3p acts as a diagnostic miRNA or a tumor suppressor mainly by suppressing FIP200 and inhibiting ATG5.

Deregulation of miR224-3p has been reported in plasma from CRC patients [50] and peripheral blood mononuclear cells from patients with non-segmental vitiligo [51]. However, besides autophagy, the function of miR224-3p in cancer is not clear. In the present study, we observed that miR224-3p suppressed GBM cells proliferation and increased hypoxia-induced apoptosis. This phenomenon may be related to the inhibitory role of miR224-3p in autophagy. However, the down-regulation of FIP200 has been shown to both induce apoptosis and inhibit the proliferation of cancer cells [26]. Therefore, the mechanisms underlying the physiological functions of miR224-3p should be further illustrated.

In conclusion, we identified hypoxia-down-regulated miR224-3p as a novel inhibitor of hypoxia-induced autophagy in GBM by directly targeting ATG5 and FIP200 (Figure 9B). MiR224-3p overexpression markedly reversed hypoxia-induced autophagy. Based on previous studies and our recent research, we propose that hypoxia-induced changes in miRNAs play important roles in the malignant progression of GBM by regulating autophagic activity. Notably, overexpression of miR224-3p suppresses tumor growth both *in vivo* and *in vitro*, implicating an anti-tumorigenesis role for miR224-3p in GBM. Further work is required to define the mechanisms of hypoxia in down-regulating miR224-3p and to illuminate whether miR224-3p could be a diagnostic marker for glioma.

## MATERIALS AND METHODS

### Ethics statement

The study has been conducted in accordance with the ethical standards and according to the Declaration of Helsinki and national and international guidelines. This study was approved by the Institutional Review Board of Shandong University. Written informed consent was obtained from all patients, and the hospital ethics committee approved the experiments.

### Tissue samples and cell lines

Human glioma cell lines (U251and U87) were purchased from the Chinese Academy of Sciences Cell Bank (Shanghai, China). Both U251 and U87 cells had been recently authenticated based on cross species checks, DNA authentication and quarantine. The cell lines were grown in Dulbecco's modified Eagle's medium (DMEM, SH30022.01B, Hyclone, UT, USA) supplemented with 10% fetal bovine serum (10082147, Gibco, MD, USA) in a humidified incubator with 5% CO2 at 37°C. Hypoxic treatment was performed by incubating cells in an incubator chamber flushed with a gas mixture containing 1% O2, 5% CO2 at 37°C. Six normal brain tissues were collected from patients undergoing internal decompression surgery following severe traumatic brain injury. Thirty human glioma tissues, including fourteen low-grade glioma tissues (four grade-I tumors and ten grade-II tumors) and sixteen high-grade glioma tissues (five grade-III tumors and eleven grade-IV tumors) were obtained from the Department of Neurosurgery, Qilu Hospital of Shandong University. Glioma specimens were verified and classified by two experienced clinical pathologists according to the WHO standard classification of tumors.

### Chemical reagents, miRNA mimics, miRNA inhibitors, plasmids, and transfections

Bafilomycin A1 (BAF) and Bis Benzimide H33258 (Hoechst 33258) were obtained from Sigma-Aldrich, USA (B1793, D8030). DMOG was purchased from Biovision Incorporated, USA (2216-5). All miRNA (miR224-3p) mimics, inhibitors, negative control and miRNA inhibitor N.C were designed and purchased from Gene Pharma (Shanghai, China). The miRNA oligo sequences are listed in Supplementary Table S1. The pcDNA3.1-FIP200 and pcDNA3.1-ATG5 plasmids used for co-transfection were constructed and validated by DNA sequencing in Gene Chem (Shanghai, China) and Gene Pharm (Shanghai, China) respectively. Cell transfection and co-transfection experiments were performed with nucleic acids using Lipofectamine 2000 (11668-019, Life Technologies, CA, USA) according to the manufacturer's instructions.

### MiRNA microarray

Cultured normoxic and hypoxic U251 cells were prepared with three samples for each condition as the biological replicates. The total mRNA was extracted for the microarray assay (KangChen Bio-tech, Shanghai, China). Total RNA was harvested using TRIzol (15596-026, Invitrogen) and the miRNeasy mini kit (QIAGEN) according to manufacturer's instructions. After RNA quantity measurement using the NanoDrop 1000, the samples were labeled using the miRCURY™ Hy3™/Hy5™ Power labeling kit (Exiqon) and hybridized on the miRCURY™ LNA Array (Exiqon, v.18.0). Scanning was performed with the Axon GenePix 4000B microarray scanner. GenePix pro version 6.0 was used to read the raw intensity of the image. Background subtraction and normalization were performed. After normalization, significant differentially expressed miRNAs were identified through Volcano Plot filtering. Finally, hierarchical clustering was performed to show distinguishable miRNA expression profiling among samples.

### RNA isolation, reverse transcription, and quantitative real-time PCR

Q-PCR was conducted to measure the expression levels of U251 and U87 cells. Total RNA was isolated using RNAiso Plus (9108, Takara). Total RNA (0.5–1 μg) was reverse-transcribed with miR224-3p, miR210 stem-loop and U6 snoRNA RT primers (RiboBio, Guangzhou, China) using a ReverTraAce qRT-PCR kit (FSQ-101, Toyobo) according to the manufacturer's protocol to synthesize cDNA. Real-time PCR was performed using a SYBR Premix Ex TaqTM Kit (QPK-201, Toyobo) with miR224-3p, miR210 and U6 snoRNA primers (miRQ0009198-1-1 for miR224-3p, miRQ0000267-1-1 for miR210, MQP-0201 for U6, RiboBio) as previously described [21, 52]. The reactions were performed using a Lightcycler 2.0 instrument (Roche Applied Science). mRNA levels were normalized to GAPDH. U6 expression was used as the endogenous control for miRNA level. All data for each sample were collected in triplicate. The fold changes were calculated by relative quantification (2^−ΔΔCt^). The primers used in the present study are listed in Supplementary Table S1.

### GFP-LC3 stable cell lines and quantitative GFP-LC3 analysis

To obtain U251 and U87 GFP-LC3 stable cell line, we cloned HAS-GFP-LC3 to the pcDNA3.1 expression plasmids and generated lentiviruses by Gene Pharm. U251 and U87 cells were infected with the viruses and selected stable clones with G418 (GDJ958, Sangon Biotech Co. Ltd, Shanghai, China). U251 and U87 GFP-LC3 stable cell lines were transfected with miR224-3p mimics or inhibitors under hypoxia or normoxia and fixed in 4% paraformaldehyde. GFP-LC3 puncta-formation assay was determined by capturing images using Olympus microscope (DP72, Japan). Cells with more than or equal to 5 puncta were considered as GFP-LC3 puncta-positive cells. The percentage of GFP-LC3 puncta-positive cells was quantified by counting 200 GFP- LC3 stable cells.

### Transmission electron microscopy

Transfected U251 cells were fixed with 3% glutaraldehyde in PBS for 2 h, washed five times with 0.1 M cacodylate buffer, and postfixed with 1% OsO4 in 0.1 M cacodylate buffer containing 0.1% CaCl for 1.5 h at 4°C. The samples were then stained with 1% Millipore-filtered uranyl acetate, dehydrated in increasing concentrations of ethanol, infiltrated, and embedded in LX-112 medium (Ladd Research Industries, Inc.). After polymerization of the resin at 60°C for 48 h, ultrathin sections were cut a Leica Ultracut microtome (Leica). Sections were stained with 4% uranyl acetate and lead citrate, and images were obtained using a JEM-100cxII electron microscope (JEM).

### Western blot analysis

Total protein was extracted from tissues and cells using RIPA buffer (P0013B, Beyotime, Shanghai, China) with 1% phenylmethyl sulfonylfluoride, and Protein concentration was determined by the BCA method (23225, Beyotime). Proteins were separated using 10–15% SDS-PAGE and transferred onto polyvinylidene difluoride membranes (ISEQ00010, Millipore, USA). The membranes were blocked by 5% skim milk blocking buffer for 1 hour and then incubated in the primary antibodies at 4°C overnight. After washing with TBST, the blots were incubated with horseradish peroxidase-conjugated secondary antibodies at room temperature for 1 h. Finally, protein bands were visualized by enhanced chemiluminescence (ECL) (WBKLS0100, Millipore) and detected using an ECL detection system (Thermo Scientific, Beijing, China) and quantified with Quantity One software. The following primary antibodies were used: Rabbit anti-LC3B, SQSTM1, SIRT1, p-mTOR (S2448), AKT, *p*-AKT (Ser473), p-ERK1/2 (Thr202 / Tyr204) were purchased from Cell Signaling Technology, MA, USA (2775, 5114, 9475, 2974, 4691, 9271, 4377). Rabbit anti-ATG5, ATG3, ERK1/2 and mouse anti-HIF1α were purchased from Abcam, Cambridge, UK (109490, 108282, 196883, 1). Rabbit anti-FIP200 antibody was purchased from Proteintech, Wuhan, China (17250-1-AP). Rabbit anti-GAPDH antibody was purchased from Goodhere Biotechnology Co. LTD, Hangzhou, China (AB-P-R 001).

The relative integrated density values were measured based on the GADPH protein as the control.

### Immunohistochemistry and semi-quantitative estimates of immunostaining

Paraffin-embedded samples were sliced and mounted on microscopic slides. Rabbit anti-FIP200 (1:100 dilutions), anti-ATG5, anti-LC3B antibodies (1:200 dilutions) and a mouse anti-HIF1α antibody (1:200 dilutions) were used as the primary antibodies. Heat-induced epitope retrieval was performed with a microwave in 10 mmol/L citric acid buffer at pH 7.2. The samples were incubated with the antibody overnight in a humidified chamber at 4°C followed by incubation with a horseradish peroxidase-conjugated secondary antibody (ORIGEN, Beijing, China, PV-9000). Finally, 3, 3′-diaminobenzidine tetrahydrochloride (DAB) was used to reveal the signal.

The total immunostaining score was estimated using both the percentage of positively stained tumor cells and the staining intensity. The percentage positivity was scored as “0” (<5%, negative), “1” (5–25%, sporadic), “2” (25–50%, focal), or “3” (>50%, diffuse). The staining intensity was scored as “0” (no staining), “1” (weakly stained), “2” (moderately stained), or “3” (strongly stained). Both the percentage of positive cells and the staining intensity were evaluated under double-blind conditions. The immunostaining score was calculated as the percentage positive score multiplied by the staining intensity score and ranged from 0 to 9. Based on the immunostaining score, the glioma patients were divided into two groups: the low expression group (0–3) and the high expression group (4–9).

### Bioinformatics prediction and luciferase reporter assay

The targets of miR224-3p were obtained from the following target prediction programs: http://microRNA.org, miRBas, TargetMiner and DIANA-TOOLS. A 290-bp fragment of the wild-type (WT) ATG5 3′ UTR (Supplementary Table S2) containing one conserved miR224-3p binding site (position 1874–1880) or a mutant ATG5 3′ UTR sequence was cloned into the pmirGLO vector (E133A, Promega, WI, USA). For FIP200, a 290-bp fragment of the 3′UTR sequence containing two conserved miR224-3p binding sites (position 6036–6042 and 6153–6159) and a mutant sequence were cloned as for ATG5 (Supplementary Table S2). GBM cells were co-transfected with the luciferase reporters together with miR224-3p mimic or NCm using Lipofectamine 2000.

### Cell proliferation and apoptosis analysis

Cell proliferation was assessed with Cell CountingKit-8 (CCK-8; CK04-500, Dojindo, Kumamoto, Japan) and EdU incorporation (C10310-3, Ribobio, Guangzhou, China) assays. For CCK-8 assay, optical density was measured at 450 nm on a microplate reader (Bio-Rad, USA). Cell viability % = (OD_0 h, 24 h, 48 h_−OD_blank_) / (OD_0 h_−OD_blank_). EdU assay was performed according to the manufacturer's protocol. Apoptosis was examined using the Annexin V-FITC Apoptosis Detection Kit (CA001-1, Signalway Antibody, MD, USA) and analyzed by a FACScanflow cytometer (BD, USA). For Immunocytochemistry, primary antibody of anti-cleaved-caspase 3 (9664, Cell Signaling Technology) was used to detected the apoptosis of cells.

### Animal experiments

All animal experiments were performed in accordance with the NIH Guide for the Care and Use of Laboratory Animals. We performed all animal surgeries under ketamine anesthesia and took every effort to minimize animal suffering. Athymic nude mice (male; 4 weeks old; 20–30 g) were provided by Shanghai SLAC Laboratory Animal Co., Ltd (Shanghai, China). The mice were randomly divided into two groups (negative control lentivirus group, *n* = 5; miR224-3p mimic lentivirus group, *n* = 5). U87 cells (2 × 10^6^) in 100 μl of PBS were inoculated subcutaneously into the flanks of nude mice. Twenty microliters of lentivirus (1 × 10^9^ TU/ml) were directly injected into each tumor twice a week. Injections began when tumor volume reached 150–200 mm^3^. Mice were sacrificed after 2 weeks.

### Statistical analyses

Data analyses were conducted with SPSS 16.0 (SPSS, IL, USA) and GraphPad-Prism5 (GraphPad, CA, USA). Data were analyzed using one-way ANOVA, Student's 2-tailed *t* test or Fisher's exact test. Data are presented as mean ± standard deviation (SD) of three independent experiments, followed by Dunnett's test for multiple comparisons of the means. All tests were 2-tailed, and *p* < 0.05 was considered statistically significant.

## SUPPLEMENTARY FIGURES AND TABLES


