# Supplementary material for: MiR224-3p inhibits hypoxia-induced autophagy by targeting autophagy-related genes in human glioblastoma cells
Source: Oncotarget. 2015 Oct 19;6(39):41620–37. doi: 10.18632/oncotarget.5871 (PMC4747177; doi:10.18632/oncotarget.5871)
Supplement: Supplementary file 1 [file oncotarget-06-41620-s001.pdf]

SUPPLEMENTARY FIGURES AND TABLES

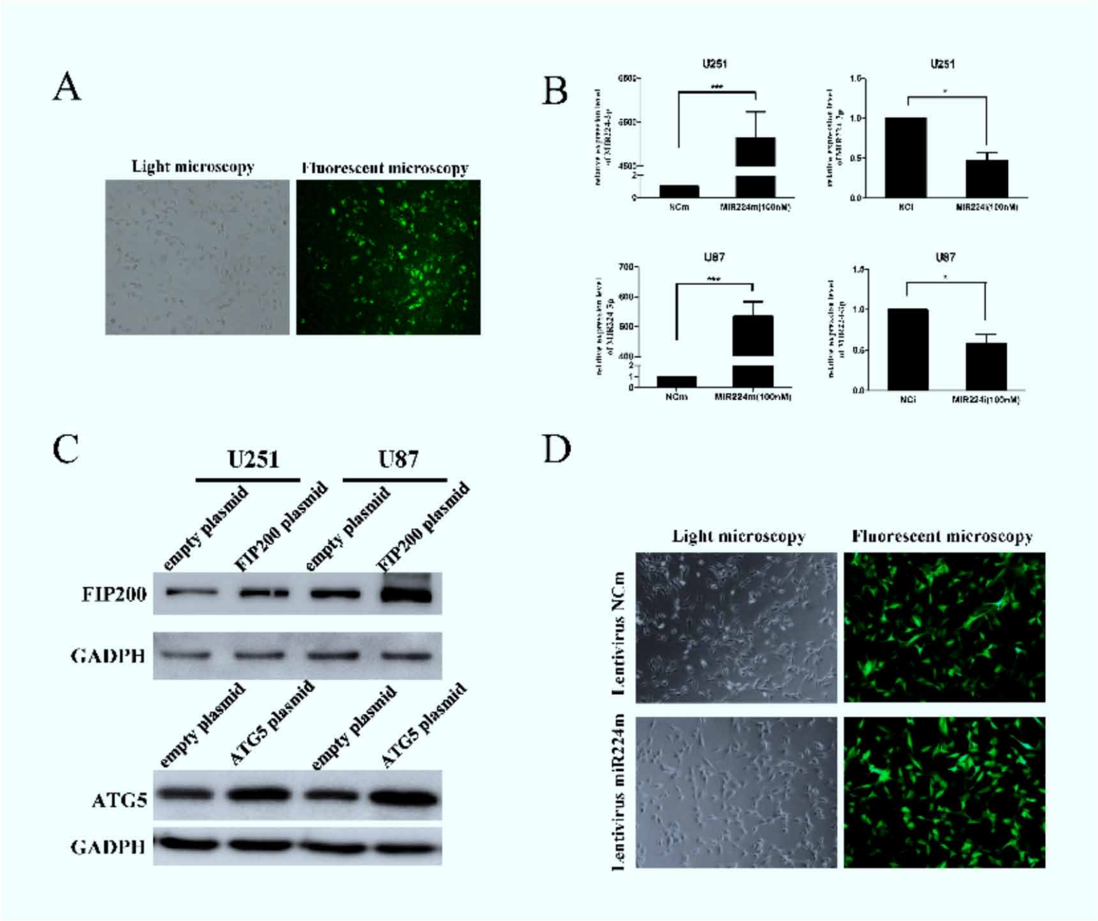

Supplementary Figure S1: The transfection efficiency of miR224-3p oligos and plasmids was high in glioblastoma cells. A. U251 cells were transfected with 100 nM FAM for 6 h, and then images were captured using a fluorescence microscope. Light microscopy, 100 × ; fluorescent microscopy, 100 × . **B.** U251 and U87 cells were transfected with miR224m, NCm and miR224i, NCi for 24 h. The cells were collected for q-PCR to quantify miR224-3p expression. The data shown are the mean ± SD of independent experiments, *n* = 3. **C.** Comparison of ATG5 and FIP200 expression between transfected empty plasmid and overexpression-plasmids cells were analyzed by Western blot. **D.** GBM cells were co-incubated with lentivirus for 72 h. Images were captured using a fluorescence microscope, 100 × . MiR224m, miR224-3p mimic; miR224i, miR224-3p inhibitor; NCm, miRNA mimic negative control; NCi, miRNA inhibitor negative control. *$P < 0.05$, ***$P < 0.001$, Student's 2-tailed *t* test.

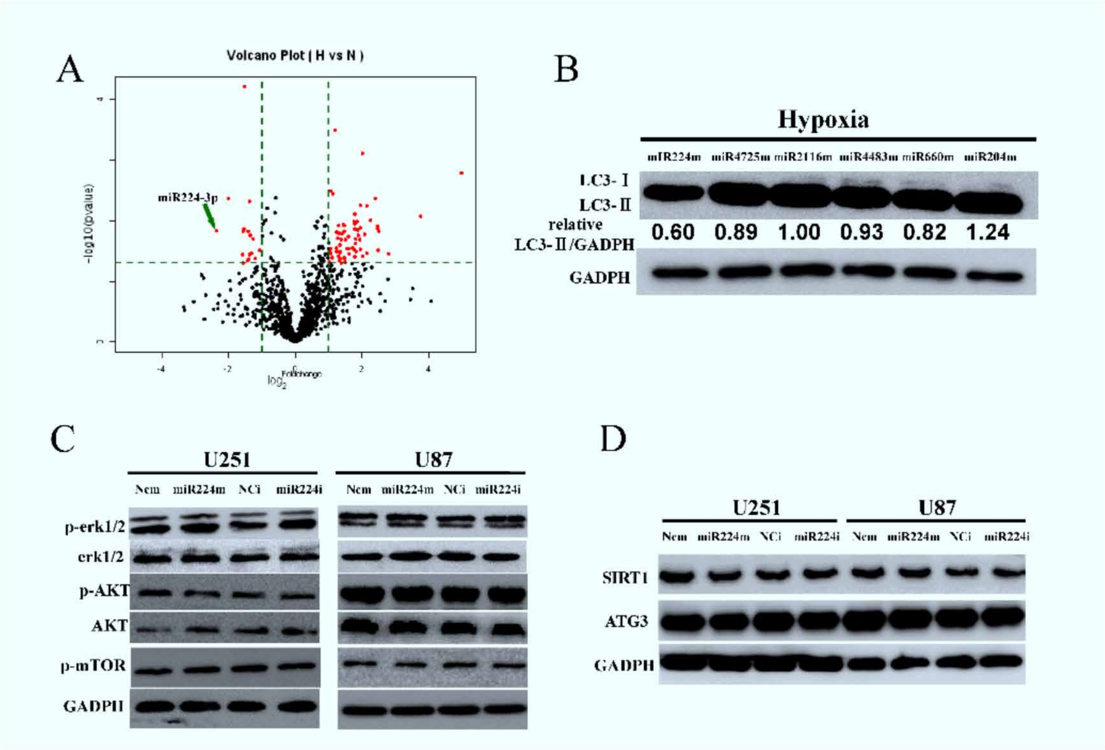

Supplementary Figure S2: miR224-3p suppresses autophagy of glioblastoma cells independent of mTOR signaling and predicted targets including SIRT1 and ATG3. A. 84 significantly dysregulated miRNAs marked with red color under hypoxia are shown on a Volcano plot. The green arrow points to the miR224-3p. **B.** U251 cells were transfected for 48 h with mimics of six miRNAs down-regulated by at least 2.65-fold in the hypoxic microarray. Then transfected cells were exposed to hypoxia for another 24 h. The cells were harvested for Western blot. The LC3B-II/GAPDH protein ratio was calculated following Quantity One analysis. **C.** and **D.** U251 and U87 cells were transfected with miR224m, NCm or miR224i, NCi and incubated for 48 h. The cells were then harvested for Western blot analysis of mTOR signaling players, SIRT1 and ATG3. MiR224m, miR224-3p mimic; miR224i, miR224-3p inhibitor; NCm, miRNA mimic negative control; NCi, miRNA inhibitor negative control.

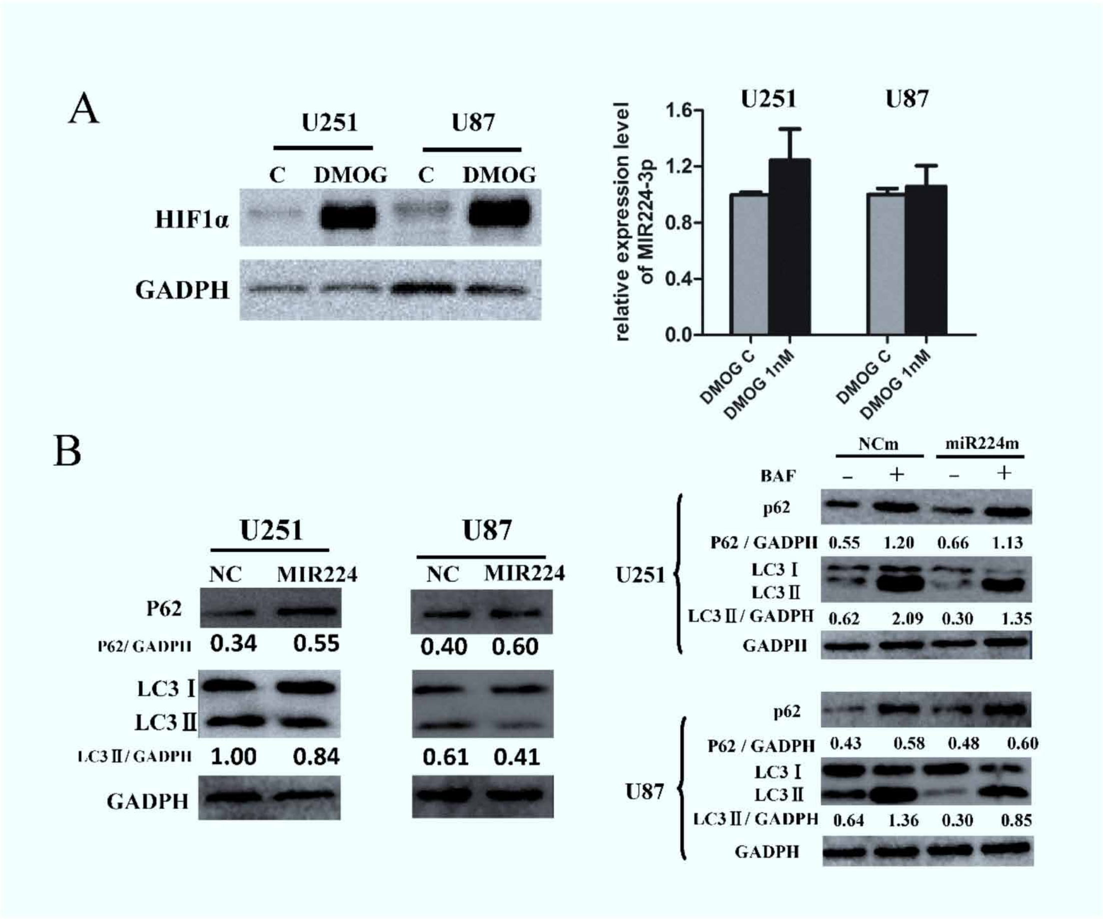

Supplementary Figure S3: Hypoxia down-regulates miR224-3p expression independent of HIF1α and miR224-3p slightly decreases GBM cell autophagic activity in normoxia. A. 1 mM DMOG was used to stabilize HIF1α for 24 h. The expression of miR224-3p was measured by q-PCR. **B.** U251 and U87 cells were transfected with miR224m (100 nM) or NCm and BAF (20 nM) were applied to the medium for 24 h at 24 h post transfection. LC3B, p62 and GAPDH levels were determined by Western blot. Student's 2-tailed *t* test.

Supplementary Table S1: Primers and RNA oligo used for the present study

Primers for q-PCR	Forward primer sequence (5′-3′)	Reverse primer sequence (5′-3′)
ATG17(FIP200)	GAAAGAGCTTGCTCAGGGATT	TCATCAACTGATTTGCGTGACT
ATG5	AAAGATGTGCTTCGAGATGTGT	CACTTTGTCAGTTACCAACGTCA
ATG3	GATGGCGGATGGGTAGATACA	TCTTCACATAGTGCTGAGCAATC
SIRT1	TAGCCTTGTCAGATAAGGAAGGA	ACAGCTTCACAGTCAACTTTGT
ATG12	TCTCCCGAGGTCTGTAGTCG	AGGGGGACGCAATTACACAG
ATG14	GCGCCCAAATGCGTTCAGAG	AGTCGGCTTAACCTTTCCTTCT
ATG10	CCCTTGGATGATTGTGAAGTGA	CTGTAGCAGTCGCATCTTATAGC
AMPKα1	TTTGCGTGTACGAAGGAAGAAT	CTCTGTGGAGTAGCAGTCCCT

RNA oligo	Sequence (5′-3′)
MIR224-3p mimics	AAAAUGGUGCCCUAGUGACUACA
	UAGUCACUAGGGCACCAUUUUUU
MIR224-3p inhibitor	UGUAGUCAUAGGGCACCAUUUU
Negative control	UUCUCCGAACGUGUCACGUTT
	ACGUGACACGUUCGGAGAATT
inhibitor NC	CAGUACUUUUGUGUAGUACAA

Supplementary Table S2: ATG5 and FIP200 3′UTR

**ATG5 3′UTR:**
GGATCAACTATTTGCCTGAACAGAATCATCCTTAAATGGGATTT
ATCAGAGCATGTCACCCTTTTGCTTCAAT
CAGGTTTGGTGGAGGCAACCTGACCAGAAACACTT
CGCTGCTGCAAGCCAGACAGGAAAAAGATCCATGTCAGATAA
GGCAACTGGGCTGGTCTTACTTTGCATCACCTCTGCTTTCCTCCACTGCC
ATCATTAAACCTCAGCTGTGACATGAAAGACTTACCGGACCACTGAAGGT
CTTCTGTAAAATATAATGAAGCTGAAACCTTTGGCCTAAGAAGAAAATGG
AAGTATGTGCCACTCGATTTGTATTTCTGATTAACAAATAAACAGGGGT
ATTTCCTAAGGTGACCATGGTTGAACTTTAGCTCATGAAAGTGGAAACAT
TGGTTTAATTTTCAAGAGAATTAAGAAAGTAAAAGAAAATTCTGTTATCA
ATAACTTGCAAGTAATTTTTTGTAAAAGATTGAATTACAGTAAACCCATC
TTTCCTTAACGAAAATTTCCTATGTTTACAGTCTGTCTATTGGTATGCA
ATCTTGTAACTTTGATAATGAACAGTGAGAGATTTTTAAATAAAGCCTC
TAAATATGTTTTGTCATTTAATAACATACAGTTTTGTCACTTTTCAAGT
ACTTTCTGACTCACATACAGTAGATCACTTTTTACTCTGTGTTACCATTT
TGACTGGTCGTCATTGGCATGGGGTGGATATAGGGCATAGGATTACTT
GTCTCAGAAGCTGTCATAGAATTTCTTGCTGCCAATTAAAAAACCTGTG
TTCTTTACACACTACACGTATAAATATTGTAACTGTTCATCTTTGTTGT
TTTATCACTGTAAGCCTGTCAAATCATAGTATCCTAAGCATCTGTAAAT
GCTAATTTTGCATTTTTGGAAAAACCCATTCCTTCCAAGCTAGTGTTTT
TCATTGGCTCCAGGTCTAATTTTTCACTGTGGTCCCTGGCAGCCAGTCTT
TTGAAGTTTAAAGATTACCTGTCTCTTGACTGCAGTACCTTTTCTTTAAT
TTTTACCAAAAATATCCAGAGGTTACTGGAGTTCTTATTCAATATAAGGA
AAGTTTGCTGCACTTTATTACCAAGCCTCTGGGATTTTACCAGTCAAACA
TATTTGTGCATTACATTTCATTTCTTGTGAGCTAGCTGGCTGTCCATATT
GAATGTTGACCCATTTGAGTACGCTAAAAGGCTTACAGTATCAGACACGA
TCATGGTTTTAGATCCCATAATAAAAATGAATGTTTTTCTTATAAAAAAT
TATACAAATGCTGAAGTGAGATTCTACTATTGTTCATTGCTTCCTTTTCT
TTTTCCTTTTGCGATTTTCACTGATTAATAGCACATTTCTTCACAAAATT
AGATAAAGTTGGTCAAAGACCAGATATTCTGGAATGGAAATTGTAAAGCT
TAATCAAAAAGAATAGCCAGTACAGCATACAATCTCAGAAACTTAGAAGC
AAGTAGAAAATAATTGGTTGATGTAAACGAAAGTGCCATTTTAGTAAAGG
CAGGAAAAAAATAGCAATATTTGAGTTATGTAAGGATAAAAAATCCACTG
ACTTGTATTTTTGCACAAGAGGCTGGTCTGAATATGATTGTTCACATTAA
GAGTGTTTATTCGTCGGTTCATTTTGGGGATTTTCCCCCTTGATGTTTTG
ACAGATTGAAGTGAGCTTTAGTGAGCAAAAGGATCAGAATGCAGGGAACA
CTAAGCTGTGATGAAGAAAGTGTGGTAAAAAGCCAGAGTAGTTTTATACA
GACAAAACCAGTGTCAGGCCTTTGCAGTAGGCTTGAGTGAACTTCTGATC
TAGATTTGAAAGTAAATTTTATGAAGACATTGCCCATTTTTACTTCCTCA
TTCATTATTGTACCAGCATCATAGCTTTATTACTCTAATCCCAGGTAAGT
CAAGCCTACAATGCCCTAGAGGAAGAGTAAAACCAGAAATTCATGCTGGC
TTAAATAATCTATTTTTGTTTCTTTTCATTTGAATATTTAAATTTTATGG
TTTATTAAAAAATTAAATAAAAAAAAAAAAAAAAAA

(*Continued*)

**FIP200 3′UTR:**
CTTATGGACAAAATTAATACATTCTATGACATTTTTTTCTGATTTGTCCT<br>GCAGTGCTCATTCATCACTCCAAAAACAGCAGGCCATCTTTTTATGCAAA<br>AGTCAGCGTGACAATATACTTCACTGGTGTACATCGTTTACTTTTTAACT<br>GGCTTCATTTTAGGAATAATAAATTCATCAGAATCCTTGGCTGAATTAAA<br>ATGGTTTTTGTTTTTTGGTTTTTTTTTTTACCCAGACAACTCTAGAAATG<br>CGGACCA AACTACTTCATTTTCTCAAAGGGCATACCTTGTGCATTGTGGCTTATGAT<br>GAGCCATATTAATTGCCTGTTAAATATACACTAGCTTGAACTTAGATGTT<br>AAATGTTATTATTACCAGCATTTGTCCTTTTGTGAAATCAGTATCAGAAT<br>ACTTGCACTCTTTAACACATTCTTTATAAAATGTATAAATTATTCAGAAC<br>TATTTAAAATAAGAGGAGTGTTATTGCATGCTGATAATCATTTTGAGTTT<br>GCCTCAGTAGATACTAAAGCAAATTGTTTCAGTTTTTTTAAATGCCCTTT<br>GATGTTTCAAAAAAAAAAAAGGAACTGTAATTTGATTGACTGATTTTAAGA<br>TCAGCCATAAGTAATCAGCAATCTTCAAAAGCACTTTCAGTGGATTGGTC<br>ATCTGGGTTCTAAAGGGAAGAGTCTGTGCTACTAACCATTTCAAATGCAG<br>ACTCAAACCTTCCCAACATCTTTATGACTCTAGAATAATCATATTGATG<br>AAATCGTAATTCATGGTTGAGTTTCAGAACAAAAGATATTCATTGCACAT<br>TAACCATTTAGAGGTCATTTAAATAACAAAATATTGTATTGTAAAAGAAC<br>TGTACAATTTTAAAACAATAAAGATTTGAACCTGTAAATGTGTGTGCCT<br>TTTAAAGAAGGATACATTTTTAATATATTTGAGTGATTGCTGGGAAGTGT<br>GAAAATATTGT TATGTATCATATCAAAGAGAAACATGTTTATTACAAAAATGTTCTTTAAC<br>TATATACTATGTAACAGGGTAAACAGTGTTATGTAGAATAGAATTGTGTA<br>AACTAGATCTTTAGAGAAGTTGCCATTGAGCAAAGTTATTTAAATGAGTT<br>AGTTGAGTTGGATGAGAATTGTTTGAGGTTTGTTGCTAGAGAACAATAAT<br>AAAATAATTCTTTTTCAGAAAATATTTAATTTCTTCATAAAAATAAGTTA<br>AATATTTTTTTAAATATGTATATCTAATAGTACAAAATGGAATAAACATC<br>ATAGTGTATAGAAAACTGAATTTGACAAGTTAATGAATAAATGAACAAAT<br>GATTTCAAAA

Red words: Cloned sequences
Yellow background: Conserved binding sites of MIR224-3p
